# Transscleral diffusion of ethacrynic acid and sodium fluorescein

**Published:** 2007-02-22

**Authors:** Cheng-Wen Lin, Yong Wang, Pratap Challa, David L. Epstein, Fan Yuan

**Affiliations:** 1 Department of Biomedical Engineering, Duke University, Durham, NC; 2 Department of Ophthalmology, Duke University, Durham, NC

## Abstract

**Purpose:**

One of the current limitations in developing novel glaucoma drugs that target the trabecular meshwork (TM) is the induced corneal toxicity from eyedrop formulations. To avoid the corneal toxicity, an alternative approach would be to deliver TM drugs through the sclera. To this end, we quantified ex vivo diffusion coefficient of a potential TM drug, ethacrynic acid (ECA), and investigated mechanisms of ECA transport in the sclera.

**Methods:**

An Ussing-type diffusion apparatus was built to measure the apparent diffusion coefficient of ECA in fresh porcine sclera at 4 °C. To understand mechanisms of ECA transport, we quantified the transscleral transport of a fluorescent tracer, sodium fluorescein (NaF), that has a similar molecular weight but is more hydrophilic compared to ECA. Furthermore, we developed a mathematical model to simulate the transport processes and used it to analyze the experimental data. The model was also used to investigate the dependence of diffusion coefficients on volume fraction of viable cells and the binding of NaF and ECA to scleral tissues.

**Results:**

The diffusion coefficients of ECA and NaF in the sclera were 48.5±15.1x10^-7^ cm^2^/s (n=9) and 5.23±1.93x10^-7^ cm^2^/s (n=8), respectively. Both diffusion coefficients were insensitive to cell shrinkage caused by ECA during the diffusion experiments and cell damage caused by the storage of tissues ex vivo before the experiments. Binding of ECA to scleral tissues could not be detected. The apparent maximum binding capacity and the apparent equilibrium dissociation constant for NaF were 80±5 mM and 2.5±0.5 mM (n=3), respectively.

**Conclusions:**

These data demonstrated that ECA diffusion was minimally hindered by structures in the sclera, presumably due to the lack of cells and binding sites for ECA in the sclera.

## Introduction

Glaucoma is one of the leading causes of blindness in the world [1,2]. One of the main risk factors for primary open-angle glaucoma is elevated intraocular pressure (IOP); and lowering IOP is the only effective therapy currently available [3]. It has been known for nearly 40 years that the locus of this hydrodynamic disease resides in the trabecular meshwork (TM) outflow pathway [4-6], but there are currently no specific treatments targeted to the TM [2]. Most potential TM drugs, e.g., ethacrynic acid (ECA) that influences the cytoskeleton in TM cells, are currently delivered topically, which is limited by the induced corneal toxicity observed at the drug dosage required for adequate transport across the cornea [7,8].

One alternative approach to TM drug delivery, which can avoid the cornea toxicity, would be to deliver drugs through the sclera. To this end, we quantified the transscleral permeability and diffusion coefficients of ECA in fresh porcine sclera. To determine mechanisms of ECA transport in the sclera, we also investigated the transscleral transport of sodium fluorescein (NaF), a highly hydrophilic molecule, and compared the results with those of ECA. Although their molecular weights are similar (i.e., 303 versus 376), we observed that the porcine sclera was more permeable to ECA than NaF. In addition, the diffusion coefficients of ECA and NaF were insensitive to changes in volume fraction of viable cells in the sclera. To quantitatively understand mechanisms of the difference in the diffusion coefficients between ECA and NaF, we developed a mathematical model to simulate diffusion and binding of these molecules in the sclera, and compared the simulation results with the experimental data.

## Methods

### Preparation of fresh porcine sclera

**Figure 1:**
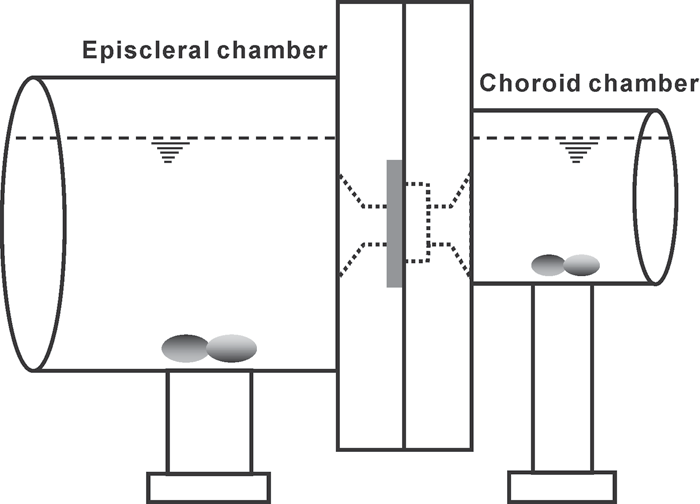
Schematic of the Ussing-type diffusion apparatus. The sclera was mounted between the two chambers with the intraocular side of the sclera facing the choroid chamber (8 ml in volume) and the episcleral side facing the episcleral chamber (30 ml in volume). The solutions in both chambers were stirred continuously by magnetic bars and the temperature was maintained at 4 °C in all experiments.

Freshly enucleated porcine eyes were obtained locally from a slaughterhouse and stored at 4 °C. The time interval between porcine sacrifice and the use of eyes in transport experiments was approximately 8 h. During the experiments, the adherent episcleral tissues were carefully removed and the eyes were bisected along the sagittal plane. The cornea, limbus, optic nerves, choroid, and retina were thoroughly excised with minimal damage of the sclera. The prepared scleras were used either immediately in our experiments or stored for up to 3 days at 4 °C. The thickness of the sclera was measured using a digital caliper. A circular piece of the sclera, approximately 11 mm in diameter, was dissected using sterile curved scissors and immediately mounted in a diffusion apparatus (Figure 1). A rubber O-ring with an inner diameter of 6.93 mm was then placed on the episcleral side of the tissue to prevent leakage of medium through the junction of the two chambers in the apparatus. The data were discarded if any leakage was observed.

### Volume fraction and viability of cells in the sclera

The methods for quantification of volume fraction and viability of cells in biological tissues have been described in previous studies [9-11]. In brief, 50 mg of scleral tissue was treated with 150 μl protease (Sigma, St. Louis, MO) in 1,350 μl DMEM at 37 °C for 1 h. The suspension was centrifuged at 3,000 rpm for 3 min to remove the protease. The tissue pellet was then resuspended in 1,350 μl DMEM containing 300 μl collagenase (type IA; Sigma) at 37 °C until they were fully digested. The numbers of dead and viable cells in the suspension were quantified using the LIVE/DEAD assay (Molecular Probes, Eugene, OR) [9-11]. The viability of cells was defined as the number of viable cells divided by the total number of cells (viable + dead) observed under a fluorescence microscope. The volume fraction of cells was calculated as (πd_cell_^3^/6)N_cell_/(C_tissue_/ρ_tissue_), where d_cell_ is the cell diameter and ρ_tissue_ is the mass density of scleral tissues. They were assumed to be 10 μm and 1 g/cm^3^, respectively. N_cell_ is the number of cells per unit volume of the suspension measured using a hemocytometer, and C_tissue_ is the tissue weight per unit volume of the suspension that was equal to 30 mg/ml in our experiments. All measurements were repeated four times.

### Dialysis experiment

The equilibrium dialysis method was used to investigate nonspecific binding of NaF and ECA to scleral tissues. In the experiment, fresh porcine scleral tissues were first digested completely using the method described above for cell viability, except that the DMEM was replaced by phosphate-buffered saline (PBS) containing Na_2_HPO_4_ and KH_2_PO_4_. To minimize the binding of NaF and ECA to the dialysis membrane, we used two different membrane materials: cellulose ester for NaF and regenerated cellulose for ECA (Float A Lyzer; Spectrum Laboratories Inc., Los Angeles, CA). The molecular cutoff sizes of these membranes were 2,000 Da and 3,500 Da, respectively. The membranes were dispensed overnight in deionized water at 4 °C to remove the preservative (i.e., sodium azide). NaF (or ECA) in PBS and the solution of digested tissues were prepared and then mixed at 30 min before each dialysis experiment to ensure that the binding between NaF (or ECA) and the tissues had reached equilibrium. The total volume of the mixture was 1,650 μl, in which the concentration of digested tissues was 30 mg/ml and the concentration of NaF (or ECA) varied in experiments. They were 25, 50, 75, 100, 200, 400, 1000, 2000, or 5000 μM for NaF and 0.05, 0.1, 0.3, 0.6, 1, 2, 5, 10, or 16.5 mM for ECA. The mixture was added into the dialysis tubing that was placed in 25 ml of PBS stirred with a magnetic bar at 4 °C for 72 h when the concentration equilibrium across the dialysis membrane was reached for the unbound NaF and ECA molecules. This equilibrium time (i.e., 72 h) was predetermined in a control experiment in which the scleral tissues were absent in the dialysis tubing and the concentrations of NaF (or ECA) in both the tubing and the buffer were monitored intermittently until they were approximately equal to each other. At equilibrium, the concentration of unbound NaF (or ECA) in the dialysis tubing was presumably uniform and equal to the concentration of NaF (or ECA) in the buffer outside the tubing.

The concentration of NaF in the buffer and the total concentration of NaF (bound and unbound) in the dialysis tubing were measured using the fluorescence microscopy technique described below in the diffusion experiment. The concentrations of ECA in the buffer and the dialysis tubing were measured using an HPLC method [12], in which the mobile phase, consisted of H_2_O, acetonitrile, and acetic acid with a volume ratio of 50:50:1, was injected into an HPLC column (Inertsil ODS-3 5U, 4.6 mm i.d.x250 mm; Varian Inc., Palo Alto, CA) at the flow rate of 1.0 ml/min. All sample solutions were filtered through a 0.20 μm syringe filter (VWR, West Chester, PA) before injecting into the HPLC system. The volume of injection was 40 μl and the running time was set at 40 min. The absorbance of samples at 230 nm was converted to the ECA concentration via a linear calibration curve. It was possible that some digested tissues were filtered out by the syringe filter before injecting into the HPLC system. However, the amounts of NaF and ECA bound to cells in the sclera were negligible compared to those bound to extracellular matrix since the volume fraction of cells was <1% in the sclera (see the data in the Results section). To determine the amounts of matrix molecules that were removed during the filtering process, we performed a control experiment, in which the concentration of NaF in the dialysis tubing was measured using both the fluorescence microscopy technique and the HPLC method. We observed that these methods resulted in the same concentration of NaF, indicating that the digested extracellular matrix molecules were not filtered out significantly.

### Diffusion experiment

An Ussing-type diffusion apparatus was built to measure the diffusion coefficients of ECA and NaF in the sclera (Figure 1). The "choroid chamber," facing the intraocular side of the sclera, was filled with 6 ml of PBS. The "episcleral chamber," facing the episcleral side of the tissue, was filled with a solution (20 ml) of ECA, NaF, or a mixture of ECA and NaF, prepared at 4 °C, using PBS. The concentration was 0.6 mM for ECA and 1, 2, 4, 5, 6, or 8 mg/ml for NaF. In the mixture, the concentrations of ECA and NaF were 0.6 mM and 5 mg/ml, respectively. All solutions were freshly prepared before each diffusion experiment and the pH of these solutions was between 6.84 and 6.95. The top surfaces of solutions in both chambers were maintained approximately at the same height to avoid a hydrostatic pressure gradient across the tissue. As a result, convection of ECA and NaF across the sclera was negligible. Solutions in both chambers were stirred continuously with magnetic bars actuated at 60 rpm. All diffusion experiments were conducted at 4 °C and the concentrations of ECA and NaF in the choroid chamber were monitored intermittently for up to 14 h through the following procedures.

For ECA, samples of solutions (1 ml) in the choroid chamber were collected and placed in micro-quartz cuvettes (Semimicro-quartz with Suprasil quartz windows; Fisher Scientific Inc., Pittsburgh, PA) at 10, 20, 30, 45, 60, 90, 120, 180, and 240 min. Absorbances of ECA at 230 nm were measured at room temperature (25 °C) using a UV spectrophotometer (model 8452A Diode Array Spectrophotometer; Agilent Technologies, Santa Clara, CA). The absorbances were converted to concentrations via a linear calibration curve. After each measurement, the collected sample was placed back into the choroid chamber to minimize the disturbance of the experimental system. For NaF, samples of solutions (<1 μl) were collected from the choroid chamber every hour for up to 14 h, using glass capillaries (Microslides, 0.1x1.0 mm I.D.; VitroCom Inc., Mt. Lks., NJ), and stored in a dark environment at room temperature until measurement. Fluorescence intensities of NaF were quantified via a fluorescence microscope (Axiovert 100; Zeiss, Thornwood, NY) equipped with a photomultiplier tube (model 9658B; Thorn EMI Electron Tubes, Rockaway, NJ) and a filter set for fluorescein (Omega Optical Inc., Brattleboro, VT). The intensities were measured at three different locations along each capillary and the average intensities were converted to concentrations via a linear calibration curve. The calibration curves for both ECA and NaF were checked periodically and the changes observed were <10% in all experiments.

### Determination of apparent permeability and diffusion coefficients of ECA

After an initial time delay, we observed that the concentration of ECA in the choroid chamber was approximately a linear function of time, indicating that the flux of ECA across the sclera had reached a steady state. The slope of the curve (ΔC_t_/Δt) was determined by linear regression of the concentrations at different time points. The square of the correlation coefficient, r^2^, in the regression was >0.96. The apparent transscleral permeability coefficient, P_app_, and the apparent diffusion coefficient, D_app_, at the steady state were calculated as;

(1) $$Papp=\frac{V}{A{\text{C}}_{0}\;}.\frac{\Delta Ct}{\Delta t}$$

(2) $$Dapp=\frac{V\delta }{A{\text{C}}_{0}\;}.\frac{\Delta Ct}{\Delta t}$$

where A is the exposed surface area of the sclera, which is equal to the area surrounded by the rubber O-ring (0.377 cm^2^), *V* is the volume of solution in the choroid chamber (6 ml), δ is the thickness of the sclera, and C*_0_* is the concentration of ECA in the episcleral chamber.

### Determination of diffusion coefficient and binding constants of NaF

In contrast to the data of ECA described above, the concentration of NaF in the choroid chamber was a nonlinear function of time even after the initial time delay (see the Results section), indicating that the transport had not yet reached a steady state and that the steady-state analysis based on Equations 1 and 2 was invalid for determining the apparent diffusion coefficient of NaF. In addition, we observed in a preliminary study that NaF transport in the sclera was significantly hindered by the binding of NaF to scleral tissues. Therefore, we developed a new method to determine the diffusion coefficient and the binding constants of NaF. The new method involved two experimental groups. In the first group, we measured the concentration of NaF in the choroid chamber as a function of time, following the same procedure as that described in the previous section. In the second group, the sclera was removed from the diffusion apparatus at 6 h after NaF was added into the episcleral chamber. The sclera was mounted vertically on a specimen block and sectioned into 10 μm slices using a cryostat (HM 505E; Microm, Kalamazoo, MI). For each sclera, 200 line profiles of fluorescence intensities across the tissue were measured, using the fluorescence microscope mentioned above and a CCD camera (CoolSNAP-Pro; Media Cybernetics, Silver Spring, MD). These profiles were then averaged; and the average profile was normalized by its maximum value. The normalized profile and the temporal profile of NaF in the choroid chamber were fit sequentially by the mathematical model described below. The nonlinear curve-fitting yielded the diffusion coefficient and the binding constants of NaF.

### Mathematical model of molecular transport across the sclera

The governing equation for one-dimensional, time-dependent transport of NaF is;

(3) $$\frac{\partial Ct}{\partial t}=Dsc\frac{\partial^{2}Cf}{\partial x^{2}}0<\text{x}<\delta$$

where t is the time, x is the distance, D_sc_ is the diffusion coefficient of NaF in the sclera, δ is the thickness of the sclera, C_f_ is the concentration of unbound NaF, and C_t_ is the total concentration of bound (C_b_) and unbound NaF in the tissue, i.e.;

(4) Ct=Cf+Cb

Equation 3 is valid for diffusion in dilute solutions or tissues in which the diffusion coefficient is independent of the concentration. To verify it, we performed a preliminary experiment and observed that the temporal profile of NaF concentration in the choroid chamber was independent of the initial concentration of NaF in the episcleral chamber ranging from 1 to 8 mg/ml.

The binding process has been modeled in a previous study of sulforhodamine diffusion in the sclera [13], in which the concentration of available binding sites is assumed to be much larger than the local concentration of sulforhodamine. In this case, the binding kinetics can be modeled as a first order chemical reaction. However, we observed that the concentration of bound NaF was a nonlinear function of the concentration of unbound NaF in the solution of enzymatically digested scleral tissues (see the Results section), indicating that the local concentration of NaF was comparable to the concentration of available binding sites. Therefore, we modeled the binding of NaF to scleral tissues as a second order chemical reaction. In addition, we assumed that the binding process was much faster than diffusion, i.e., the binding reaction was at a quasi-equilibrium state [13]. Taken together;

(5) $$Cb=\frac{B\max Cf}{KD+Cf}$$

where B_max_ is the apparent maximum binding capacity and K_D_ is the apparent equilibrium dissociation constant [14]. Substituting Equations 4 and 5 into Equation 3 yields;

(6) $$\frac{\partial Cf}{\partial t}=\frac{{\left(Cf+KD\right)}^{2}Dsc}{{\left(Cf+KD\right)}^{2}+B\max KD}\frac{\partial^{2}Cf}{\partial x^{2}}0<x<\delta$$

The initial condition was C*_f_*=0 at t=0. The boundary conditions were determined as follows. The concentration of NaF in the choroid chamber was much less than that in the sclera in our experiments. Thus, it is negligible in the transport analysis (i.e., C*_f_*=0 at x=δ). There existed a concentration boundary layer (or unstirred layer) at the tissue/solution interface in the episcleral chamber. The thickness of the boundary layer in the episcleral chamber was close the thickness of sclera, which was estimated by measuring the space between the yellow solution of NaF and the surface of sclera. The Stokes-Einstein equation predicts the diffusion coefficient of NaF in water at 4 ^o^C to be 2.89x10^-6^ cm^2^/sec [15], which was at least one order of magnitude higher than the apparent diffusion coefficient in the sclera observed in this study. Therefore, the concentration drop across the boundary layer was negligible compared to that across the sclera, i.e., C*_f_*=C*_0_* at x=0, where C*_0_* is the concentration of NaF in the episcleral chamber.

Equation 6 with the initial and boundary conditions described above was solved numerically using a finite difference method. The value of δ was measured for each sclera used in our experiment. The values of D_sc_, B_max_, and K_D_ were determined by using the simulation results to fit the experimental data of NaF. The procedures for nonlinear curve-fittings are as follows. First, the predicted concentration profile at 6 h was used to fit the spatial distribution data (see the previous section) to determine the values of D_sc_, B_max_, and K_D_ in individual tissue sections from three scleras. To verify the result of D_sc_, equation 6 with B_max_ and K_D_ fixed at their mean values from three scleras was solved again to fit the temporal profile of NaF in the choroid chamber. The fitting yielded a new value of D_sc_, which was consistent with that determined by fitting the spatial distribution data (see the Results section).

### Statistical Analysis

An unpaired Mann-Whitney U test was used to compare data from different groups. The differences were considered to be statistically insignificant if p>0.05. All data are reported as mean±SD.

## Results

### Thickness of sclera and volume fraction and viability of cells

The thickness of the porcine sclera in our experiments ranged from 0.53 mm to 1.56 mm with a mean±SD of 1.01±0.26 mm (n=28). The volume fraction and the viability of cells are shown in Table 1. In fresh scleras, they were 0.8% and 95%, respectively, i.e., the volume fraction of viable cells was 0.76%. For scleras stored at 4 °C for 72 h, the volume fraction of viable cells was reduced to 0.03%, which was only 4% of that in fresh scleras. When the temperature was increased to 37 °C, more dead cells were observed in the sclera (Table 1).

**Table 1:** Volume fraction and viability of cells in scleras stored at different temperatures and for different time periods.

**Storage time (temperature)**	**Percentage of viable cells (%)**	**Volume fraction of all cells (%)**	**Volume fraction of viable cells (%)**
0 h	95.1±2.0	0.80±0.03	0.76±0.03
24 h (4 °C)	55.4±1.8	0.35±0.04	0.19±0.02
24 h (37 °C)	27.2±6.9	0.16±0.06	0.04±0.02
72 h (4 °C)	43.9±11.4	0.06±0.01	0.03±0.00
72 h (37 °C)	33.3±3.9	0.03±0.01	0.01±0.00

The data are reported as mean±SD.

### Binding of ECA and NaF to enzymatically digested scleral tissue

**Figure 2:**
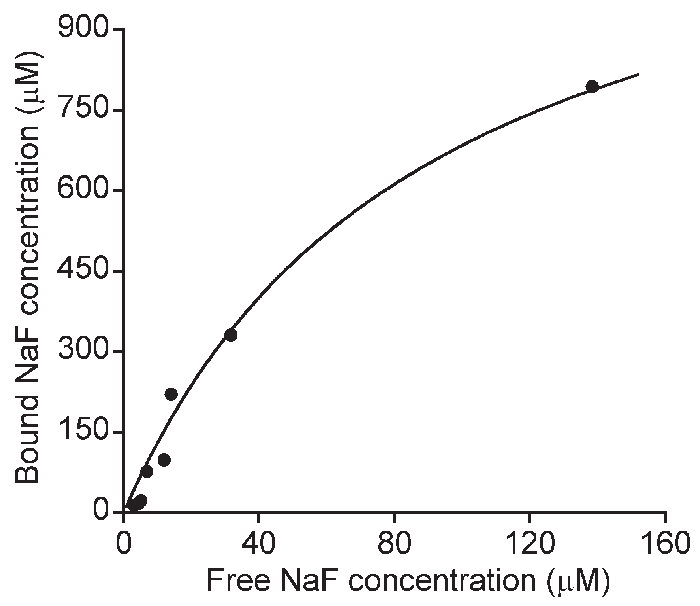
Equilibrium binding of sodium fluorescein (NaF) to enzymatically digested scleral tissue in the dialysis experiment. The concentration of bound NaF was determined as a function of the concentration of unbound (or free) NaF in the dialysis tubing. In the experiments, the concentration of scleral tissue in the dialysis tubing was 30 mg/ml. The symbol represents the data from experiments with 9 different initial concentrations of NaF in the dialysis tubing. The solid curve shows the nonlinear curve-fitting based on Equation 5, which yielded the values of B_max_ and K_D_ for NaF to be 1.44 mM and 0.11 mM, respectively.

The equilibrium binding between NaF (or ECA) and enzymatically digested scleral tissue was determined for 9 different initial concentrations using the dialysis method (see the Methods section). We measured both the total concentration (C_Td_) of NaF (or ECA) in the dialysis tubing and the concentration of the same molecule in the buffer outside the dialysis tubing (C_Ub_). At equilibrium, the concentration of unbound NaF (or ECA) in the dialysis tubing (C_Ud_) should be uniform and equal to C_Ub_. Thus, the concentration of bound NaF (or ECA) in the dialysis tubing (C_Bd_), defined as C_Td_-C_Ud_, is equal to C_Td_-C_Ub_. For NaF, C_Bd_ increased nonlinearly with increasing C_Ud_ (Figure 2). These data could be fit by equation 5, which yielded the values of B_max_ and K_D_ to be 1.44 mM and 0.11 mM, respectively. We also compared the binding of NaF to enzymatically digested versus mechanically homogenized tissues and observed no difference between these two groups in terms of B_max_ and K_D_, indicating that the enzymatic digestion did not create additional binding sites for NaF in the sclera. The value of B_max_ for NaF has to be corrected when it is used for binding analysis in the sclera, since the tissue concentration was only 30 mg/ml in the dialysis tubing but equal to the mass density in the sclera. If the mass density is assumed to be 1 g/ml, the value of B_max_ will be 48 mM in the sclera. For ECA, C_Td_ was statistically the same as C_Ub_ for all 9 different initial concentrations, indicating that binding of ECA to scleral tissues was negligible.

### Diffusion coefficient of ECA

The steady state analysis of transscleral transport resulted in the apparent permeability and diffusion coefficients of ECA, which were 42.1±8.2x10^-6^cm/s and 48.5±15.1x10^-7^cm^2^/s (n=9), respectively. These data were approximately equal to the true permeability and diffusion coefficients of ECA in the sclera since there was minimal binding of ECA to scleral tissues. When ECA diffused simultaneously with NaF through the same scleras, the permeability and diffusion coefficients of ECA were 45.4±11.3x10^-6^cm/s and 41.2±11.3x10^-7^cm^2^/s (n=7), respectively. The changes were statistically insignificant (p>0.05), indicating that NaF had no effects on the permeability and diffusion coefficients of ECA. In addition, the permeability coefficient of ECA was independent of the time period (4 h versus 3 days) and the temperature (4 ^o^C versus 37 °C) of scleras storage (data not shown), indicating that ECA diffusion was insensitive to cell damage in the tissue.

### Spatial distribution of NaF in the sclera

**Figure 3:**
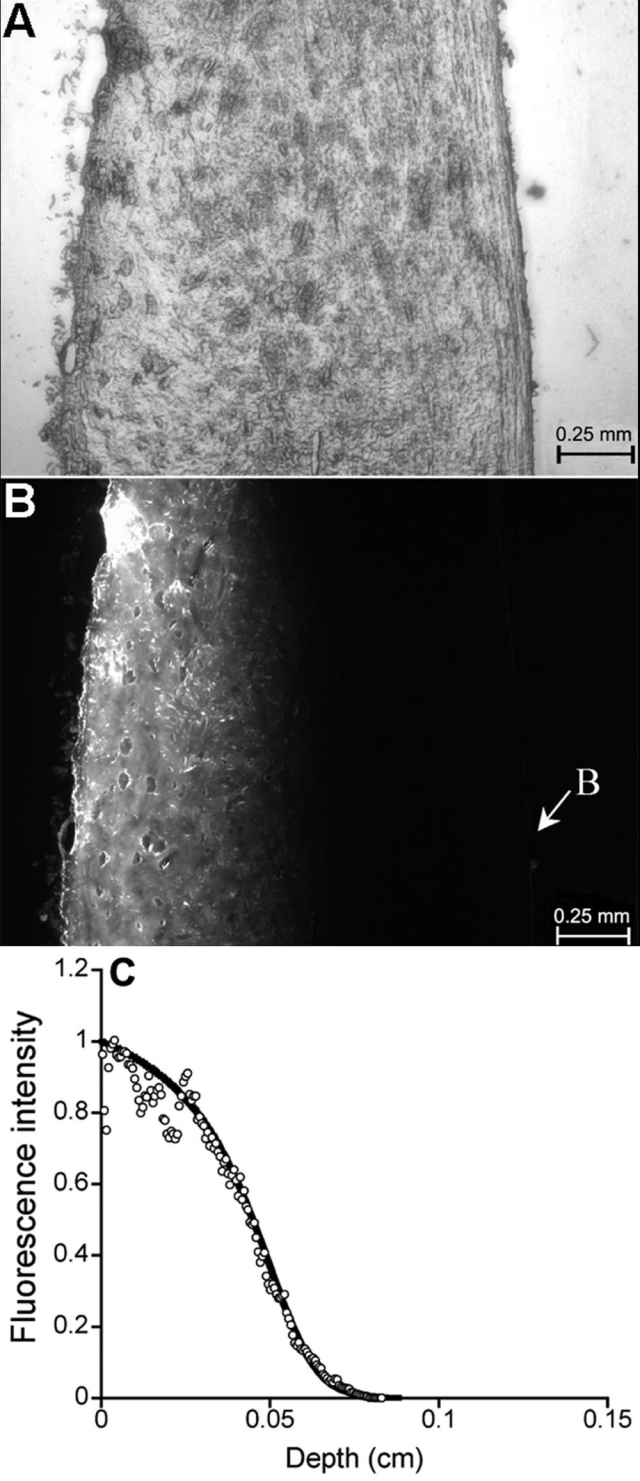
Spatial distribution of sodium fluorescein (NaF) in the sclera. The sclera was frozen and sectioned into 10 μm slices when the mixture of NaF (5 mg/ml) and ethacrynic acid (ECA; 0.6 mM) diffused through it for 6 h. Images of a typical section with trans- and epi-illuminations are shown in **A** and **B**, respectively. In **B**, B indicates the right boundary of the tissue section. The normalized distribution of the fluorescence intensity in the epi-illuminated section is shown as open circles in **C**. It was a function of penetration depth of NaF from the left to the right. The data points were fit by the normalized total concentration of NaF at 6 h in the sclera (C_t_), based on the mathematical model described in the Methods section. The result of curve-fitting is shown as the solid curve in **C**.

Typical images of a tissue section with trans- and epi-illuminations are shown in Figure 3A and Figure 3B, respectively. The transscleral profile of fluorescence intensity in Figure 3B was measured and fit by the mathematical model described in the Methods section. The result of curve-fitting is shown in Figure 3C, which yielded values of D_sc_, B_max_, and K_D_ to be 2.8x10^-7^ cm^2^/s, 80 mM, and 3 mM, respectively. The mean±SD of these parameters from three tissue sections were 3.3±0.6x10^-7^ cm^2^/s, 80±5 mM, and 2.5±0.5 mM, respectively. The value of B_max_ was 67% larger than that (i.e., 48 mM) determined by the dialysis method in digested tissues; and the value of K_D_ was one order of magnitude higher than that from the dialysis experiment. Mechanisms for explaining the discrepancy will be discussed later.

### Temporal profiles of ECA and NaF concentrations in the choroid chamber

**Figure 4:**
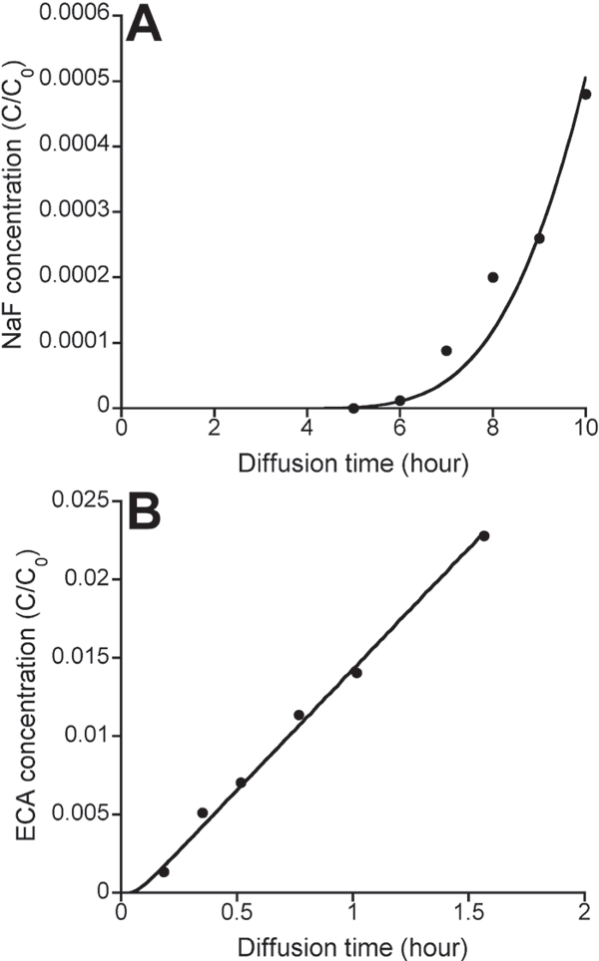
Temporal profiles of sodium fluorescein (NaF) and ethacrynic acid (ECA) concentrations in the choroid chamber. **A**: A typical temporal profile of NaF concentration in the choroid chamber. **B**: A typical temporal profile of ECA concentration in the choroid chamber. The symbols denote the experimental data normalized by the initial concentrations of NaF (5 mg/ml) and ECA (0.6 mM), respectively, in the episcleral chamber. The solid curves show the curve-fitting based on the mathematical model described in the Methods section.

**Figure 5:**
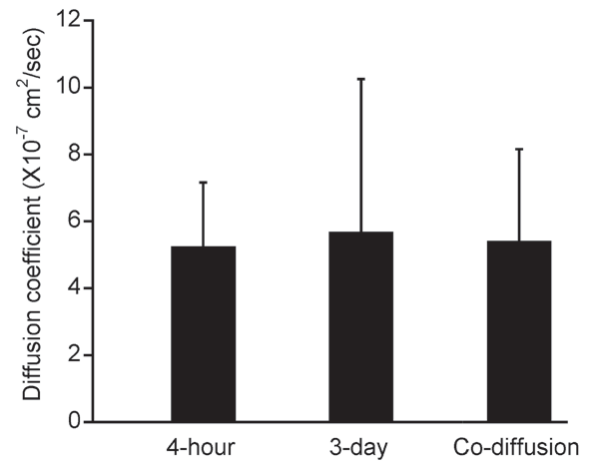
The diffusion coefficient of sodium fluorescein (NaF) in the sclera. The data were obtained through the curve-fitting shown in Figure 4A. The bar and error bar represent mean and SD, respectively. The diffusion coefficient of NaF was 5.23±1.93x10^-7^ cm^2^/s (n=8) in scleras stored at 4 °C for 4 h, 5.68±4.57x10^-7^ cm^2^/s (n=4) in scleras stored at 4 °C for 3 days, or 5.40±2.76x10^-7^ cm^2^/s (n=11) when NaF diffused simultaneously with ethacrynic acid (ECA) through the same sclera. There were no statistically significant differences between any two groups (p>0.05).

To further evaluate the diffusion coefficients of ECA and NaF in the sclera, we simulated transport of ECA and NaF across the sclera, using the mathematical model described in the Methods section and calculated the temporal profiles of ECA and NaF in the choroid chamber. These profiles were also measured experimentally; and the data were fit by the simulation results. Typical results of the curve-fitting are shown in Figure 4. In the curve-fitting, we assumed that the binding was negligible for ECA and that the binding constants, B_max_ and K_D_, for NaF in all scleras were 80 mM and 2.5 mM, respectively. Therefore, only the values of diffusion coefficients were obtained after curve-fitting. For ECA, it was statistically the same as the apparent diffusion coefficient determined by using equation 1 (data not shown), which indicated that ECA diffusion was at a steady state after a short time period at the beginning of the experiment. For NaF, the mean±SD of D_sc_ in all scleras are shown in Figure 5. In scleras stored at 4 °C for 4 h, it was 5.23±1.93x10^-7^ cm^2^/s (n=8). The diffusion coefficient was not affected by increasing in the storage period from 4 h to 3 days (5.68±4.57x10^-7^ cm^2^/s [n=4]) or co-diffusion with ECA through the same sclera (5.40±2.76x10^-7^ cm^2^/s [n=11]). These data indicated that NaF diffusion was insensitive to changes in the volume fraction of cells in tissues.

## Discussion

We investigated ECA and NaF diffusion in the porcine sclera and observed that ECA diffused approximately 8 times faster than NaF, although their molecular weights were close to each other (303 versus 376). In addition, we observed that the diffusion coefficients of ECA and NaF were insensitive to cell shrinkage caused by ECA during the diffusion experiments and cell damage caused by the storage of tissues ex vivo before the experiments, presumably due to the fact that the volume fraction of cells in the sclera was only about 1%. Therefore, the hindrance of ECA and NaF diffusion in scleral tissues was mainly caused by extracellular matrix and soluble proteins in interstitial fluid.

### Effect of binding on transscleral transport

We observed that NaF could bind strongly to scleral tissues. The binding was likely to be mediated through charge/charge interactions since fluorescein has three dissociation constants that correspond to three pK_a_ values: 2.13, 4.44, and 6.36, respectively [16,17]. At pH 7, the anionic forms of NaF may bind to positively charged extracellular matrix molecules (e.g., collagens) and proteins dissolved in interstitial fluid. The binding may effectively reduce the flux of NaF in tissues before the steady state of diffusion was reached. On the other hand, the binding of ECA to scleral tissues could not be detected. The difference in the binding between NaF and ECA could also be observed directly in terms of the time lag defined as the time period from t=0 to the time point when the concentration in the choroid chamber started to increase rapidly with time (see Figure 4). It was >5 h for NaF but 5-7 min for ECA. To quantitatively understand effects of binding on transscleral transport, we developed a mathematical model to numerically simulate the process. The simulated time lags for both ECA and NaF were consistent with the experimental data (data not shown). In the rapid increase phase of the concentration in the choroid chamber, the slope of the curve was used to calculate the apparent diffusion coefficient, D_app_. It increased with time and should eventually reach its maximum level when the diffusion is at the steady state. If the concentration boundary layer is negligible, which was the case in this study, the maximum D_app_ is equal to D_sc_, the true diffusion coefficient in the sclera. This trend in D_app_ can be predicted by equations 3 through 6. At the steady state, the binding has no effects on the transscleral diffusion.

The binding constants of NaF determined in the study depended on the experimental methods, as shown in the Results section. The values of B_max_ and K_D_ determined from the spatial distribution of NaF in the sclera were 67% and one order of magnitude, respectively, higher than those from the dialysis experiment. These discrepancies are likely to be caused by the difference in binding mechanisms between these two methods. During the diffusion in the sclera, NaF binds to fixed cationic groups on extracellular matrix as well as cationic groups on mobile proteins dissolved in interstitial fluid. If the distances between adjacent cationic groups are larger than the diameter of NaF, the binding of NaF to these groups is monovalent. Meanwhile, the binding of NaF to other cationic groups in the sclera can be either monovalent, bivalent, or trivalent since NaF has 3 anionic groups at pH>6.4 [16,17]. In the solution of digested tissues, all charged molecules are freely mobile, thereby making it easier for NaF to have bivalent or trivalent binding to cationic groups. It has been shown in the literature that the apparent equilibrium dissociation constant K_D_ for bivalent binding can be several orders of magnitude smaller than that for monovalent binding to the same substrates [18]. And the trivalent binding can further reduce the value of K_D_. The bivalent and trivalent binding will also reduce the number of bound NaF molecules and consequently the value of B_max_. Therefore, the binding valency is likely to be a mechanism to partly explain the differences in B_max_ and K_D_ mentioned above.

### Effects of hydrophobicity and charge on molecular diffusion

The 8 fold difference in the diffusion coefficients between ECA and NaF could be partly due to the differences in their hydrophobicities and charges since the molecular weights of ECA and NaF are similar (303 versus 376). NaF is soluble in water and has three pK_a_ values as discussed above. In contrast, ECA is sparingly soluble in water and aqueous acids. It has only one pK_a_ at 3.50 [19]. The pK_a_ values of these molecules indicated that the amount of the unionized form of ECA was approximately four orders of magnitude higher than that of NaF in PBS at pH 7, i.e., NaF was much more soluble than ECA in PBS. In addition, the hydrophobicity can be determined directly by the logarithm of the distribution coefficient (logD), which is equal to the partition coefficient times the fraction of the unionized form [16]. The logD of ECA between ether and water is 0.05 at pH=7.4 [19], and the logD of NaF between octanol and water at pH=7.4 was -4.28 [16]. The solubilities in ether and octanol are on the same order of magnitude for many small molecules [20]. Hence, NaF is approximately four orders of magnitude more hydrophilic than ECA. The relatively hydrophobic nature of ECA may allow it to diffuse through both intracellular and extracellular space whereas NaF can diffuse only through the interstitial fluid space. However, the volume fraction of cells in the sclera was less than 1% (see Table 1). As a result, the hydrophobicity should have minimal effects on the rate of diffusion compared to the charge of molecules. As discussed above, charge can mediate binding of NaF to immobile structures (e.g., extracellular matrix) or soluble proteins in tissues, which hinders diffusion at unsteady state. Meanwhile, the charge of solutes can affect the diffusion through mechanisms that are independent of binding. Data in the literature have shown that negatively charged molecules diffuse slower in the microvessel wall compared to neutral or positively charged molecules [21-23], presumably due to charge/charge repulsion since the basement membrane and the glycocalyx on the luminal surface of the microvessel wall are negatively charged. The charge/charge repulsion may also hinder the diffusion of NaF in the sclera.

### Comparison with previous data

The Stokes-Einstein equation predicts that the diffusion coefficients of NaF (R=0.45 nm) in water at 4 °C and 37 °C are 2.89x10^-6^ cm^2^/s and 7.28x10^-6^ cm^2^/s, respectively [15]. Ambati et al. [24] showed that the permeability coefficient of NaF through rabbit sclera at 37 °C was 8.45x10^-5^ cm/s and the thickness of the rabbit sclera was 416 mm. If one could assume that the transport experiment in [24] is at the steady state, then the apparent diffusion coefficient was 3.5x10^-6^ cm^2^/s that is 48% of that in water at 37 °C. Cheruvu and Kompella have shown that the permeability coefficient of NaF across the bovine sclera at 37 °C is 6.77x10^-6^ cm/s [25]. Treatment of the tissue with hydroxypropyl-b-cyclodextrin (HPbCD) reduces the permeability coefficient to 3.33x10^-6^ cm/s. For HPbCD treated porcine sclera, the permeability coefficient of NaF is 3.46x10^-6^ cm/s, which is approximately equal to that for HPbCD treated bovine sclera. If one could assume that the thickness of porcine sclera is 1 mm and that the transport experiment in [25] is at the steady state, then the apparent diffusion coefficient of NaF in HPbCD treated porcine sclera at 37 °C would be estimated as 3.46x10^-7^ cm^2^/s. Furthermore, one could assume that the diffusion coefficient of NaF at 4 °C is 39.7% as high as that at 37 °C, which is predicted by the Stokes-Einstein equation mentioned above, and that HPbCD treatment reduces the permeability coefficient of NaF by 50% [25]. Based on these assumptions, the estimated diffusion coefficient of NaF in untreated porcine sclera at 4 °C would be 2.79x10^-7^ cm^2^/s, which is on the same order of magnitude as that measured in our experiment (5.23x10^-7^ cm^2^/s).

In addition to NaF, the permeability coefficients of other small molecules across different scleras have been reported in the literature. Triamcinolone acetonide (TA; molecular weight, 434.5) is a steroid used for treating retinal diseases. It has been delivered through the transscleral pathway [26,27]. The permeability coefficient of TA in human sclera at 22.5 °C is 1.47x10^-5^ cm/s [26]. The permeability and diffusion coefficients of sulforhodamine (molecular weight, 558) in human sclera at 37 °C are 2.15x10^-5^ cm/s and 1.28x10^-6^ cm^2^/s, respectively [13]. The permeability coefficients of sucrose (molecular weight, 342) in human sclera at 37 °C [28] and rabbit sclera at room temperature [29] are 2.2x10^-5^ cm/s and 4.0x10^-5^ cm/s, respectively. The same parameters at 37 °C are 1.8x10^-5^ cm/s and 1.3x10^-5^ cm/s, respectively, for dexamethasone (molecular weight, 392) and 1.2x10^-5^ cm/s and 1.3x10^-5^ cm/s, respectively, for carboxyfluorescein (molecular weight, 342) [30]. Although the molecular weights of these molecules are similar to that of NaF, their permeability coefficients in rabbit sclera are smaller than that reported in [24]. Mechanisms of the discrepancy among these data remain to be investigated.

The permeability coefficient of macromolecules across the sclera has also been reported in the literature. Aihara et al. have shown that the permeability coefficient of FGF-2 (molecular weight, 17,000) across human sclera is 1.20±0.77x10^-8^ cm/s, which is only 1.7% of that of 10 kDa dextran (0.70±0.35x10^-6^ cm/s) [31]. The authors hypothesized that the difference in the permeability coefficients was caused mainly by nonspecific binding of FGF-2 to the extracellular matrix within the sclera. Exposure of sclera to latanoprost acid [31] or prostaglandins (PGs) [32] will result in an increase in the permeability coefficient of macromolecules in a dose- and time-dependent manner. The permeability increase is likely to be due to an increase in matrix metalloproteinase activities that may reduce the collagen content of the sclera [1,31,32].

### Effects of convection and temperature on molecular transport

The height of the solution in the choroid chamber was reduced by about 0.3 cm when 1 ml of ECA was removed from the choroid chamber for measuring ECA concentration, which caused a transient hydrostatic pressure difference across the sclera at a level of about 0.23 mmHg. Previous studies have shown that the pressure difference induces convective transport in the sclera but the convection of small molecules is negligible compared to their diffusion when the transscleral pressure difference is <15 mmHg [30,33]. In addition, the total period of convection in our experiments was <4.5 min (each measurement took <30 s and there were a total of 9 measurements). When compared to the total period for diffusion (240 min), the duration of the transient process accounted for <2% of the total experimental period. Taken together, the convection of ECA, induced by the transient hydrostatic pressure difference, had minimal contribution to the apparent diffusion coefficient of ECA measured in this study.

The temperature in our experiments was maintained at 4 °C in order to minimize tissue degeneration. However, it is well known that the diffusion coefficient is temperature-dependent. Therefore, the diffusion coefficient measured in our experiments needs to be multiplied by a factor in order to obtain the value of the same parameter at 37 °C. The estimated value of this factor is 2.5, as predicted by the Stokes-Einstein equation mentioned above [15].

In summary, ECA diffused 8 times faster than NaF through the porcine sclera and ECA diffusion was minimally hindered by tissue structures. As a result, transscleral delivery of ECA to the TM would be determined mainly by the concentration of ECA at the episcleral surface and the rate of ECA clearance at the TM. Results from this study may also be useful for transscleral delivery of other TM drugs, following subconjunctival administration or intrascleral implantation of controlled release devices [34,35].
